# Harem-holding males do not rise to the challenge: androgens respond to social but not to seasonal challenges in wild geladas

**DOI:** 10.1098/rsos.140081

**Published:** 2014-09-24

**Authors:** David J. Pappano, Jacinta C. Beehner

**Affiliations:** 1 Department of Anthropology, University of Michigan, Ann Arbor, MI 48109, USA; 2 Department of Psychology, University of Michigan, Ann Arbor, MI 48109, USA

**Keywords:** challenge hypothesis, loser, male contest, seasonal, testosterone, winner

## Abstract

The challenge hypothesis has been enormously successful in predicting *inter*specific androgen profiles for vertebrate males. Nevertheless, in the absence of another theoretical framework, many researchers ‘retrofit’ the challenge hypothesis, so that its predictions also apply to *intra*specific androgen comparisons. We use a wild primate, geladas (*Theropithecus gelada*), to illustrate several considerations for androgen research surrounding male contests that do not necessarily fit within the challenge hypothesis framework. Gelada society comprises harem-holding males (that can mate with females) and bachelor males (that cannot mate with females until they take over a harem). Using 6 years of data from known males, we measured androgens (i.e. faecal testosterone (fT) metabolites) both seasonally and across specific male contests. Seasonal androgen variation exhibited a very different pattern than variation resulting from male contests. Although harem-holding males had higher testosterone levels than bachelors across the year, bachelors had higher testosterone during the annual ‘takeover season’. Thus, harem-holding males did not ‘rise to the challenge’ exactly when needed most. Yet, androgen profiles across male contests indicated that both sets of males exhibit the expected fT rise in response to challenges. Results from male geladas also support the idea that the context before (e.g. male condition) and after (e.g. contest outcome) a contest are critical variables for predicting hormones and behaviour.

## 2. Introduction

For nearly a quarter of a century, the challenge hypothesis [1] has been the predominant paradigm for explaining short-term androgen responses to social challenges for male vertebrates [2,3]. This framework, originally developed for interspecific comparisons among temperate birds [1], predicts that social challenges associated with mating effort cause circulating androgens to rise to a physiological maximum—far surpassing levels necessary for homeostatic function or basal reproductive physiology (e.g. spermatogenesis, libido, mating behaviour). Yet, because high androgen levels come at the expense of paternal care [4,5] and survival [6,7], the challenge hypothesis proposes that (where necessary) androgen modulation is extremely flexible. Thus, androgen levels can be up- and downregulated by the hypothalamic–pituitary gonadal axis as necessitated by competing mating and parenting demands (‘androgen responsiveness’). It follows, then, that androgen responsiveness should track both the breeding season (of the individual) and the social system (of the species). For the most part, this broad pattern has been upheld using interspecific comparisons of seasonal androgen profiles in birds and fishes [2,3], but see [8], although support deriving from mammalian species has been less conclusive [3].

Perhaps because the field of behavioural endocrinology generally lacks unifying theoretical hypotheses (for a few elegant exceptions see [9–11]), the challenge hypothesis framework has been called upon to explain almost any androgen variation in males, even in situations only tangentially related to social challenges (e.g. life-history androgen variation in primates [12]). We argue that this one-size-fits-all application of the challenge hypothesis to androgen research has placed unnecessary (and sometimes restrictive) boundaries on how we think about androgen variation in competitive situations. To be clear, our purpose is not to challenge the challenge hypothesis. Rather, we seek to expand the hormonal and behavioural variables that researchers report in their androgen research. There are (at least) three ways that researchers can extend their analyses of androgens and behaviour surrounding male contests.

### 2.1 Seasonal versus social challenges

First, in its original form [1], the challenge hypothesis did not distinguish between elevations in androgens *in preparation* for challenges from those *in response* to challenges. The term ‘androgen responsiveness’ suggests that we should be concerned with only the immediate neuroendocrine changes that follow male contests (hereafter, ‘social challenges’). Yet, a majority of studies claiming to support the challenge hypothesis draw their evidence from *seasonal* changes in testosterone levels [13]. These studies demonstrate that seasonal periods of territory establishment or mate guarding are associated with higher androgens than other times of the year (hereafter, ‘seasonal challenges’). Yet, in the absence of detailed behavioural data across the breeding season, seasonal measures may not necessarily result from an increase in social challenges. Indeed, other factors could easily be responsible for the rise in androgens, such as increased food availability and improved body condition [14]. Thus, it is not clear whether both social and seasonal hormone changes represent the same flexible neuroendocrine response to male contests.

To address this problem, Goymann *et al.* [13] examined all avian studies that report changes in androgens in response to a social challenge (i.e. an experimental or observed male contest) or a seasonal challenge (i.e. a known breeding season) and found that many species demonstrated unexpected differences in these two measures. For example, male blue tits (*Cyanistes caeruleus*) demonstrated a dramatic seasonal increase in circulating testosterone during the breeding season. Yet, when these same males were challenged with a simulated territorial intrusion, they failed to show the expected rise in testosterone [15]. Indeed, more than half of the bird species investigated showed the expected increase in testosterone to seasonal challenges, but failed to uphold the predictions of the challenge hypothesis when faced with rival challenges via simulated intrusions [16]. Although the reasons for these discrepancies remain a puzzle, these examples highlight the importance of distinguishing these two different situations.

### 2.2 A participant's perspective of the challenge

Second, the predictions of the challenge hypothesis do not consider whether individual males anticipate the upcoming challenge or how they interpret its outcome (reviewed in [17]). Arguably, the most important contextual piece of information regarding a social challenge is whether a male wins or loses the contest (or, more importantly, whether he perceives the encounter as a win or a loss). Yet, because the challenge hypothesis was originally formulated for comparing androgen modulation across *species* (not individuals), its purpose was to identify species-typical neuroendocrine responses to challenges, not individual differences to context-specific challenges. Therefore, we argue that research on *intraspecific* responses to challenges needs to consider the context of the interaction, including contest-specific variables (e.g. whether a male is on his ‘home turf’, is playing offence or defence, or has recently won or lost a contest [18]) as well as the outcome of the contest for each contestant [11]. Research on the ‘winner/loser effect’ suggests that changes in androgens across a social challenge depend entirely on the outcome, with androgens typically rising in winners relative to losers [19]. Furthermore, the increase in androgens in winners enhances perceived fighting ability and promotes engagement in future dominance contests, whereas the decrease in losers promotes withdrawal from such contests [11]. This underscores the well-known, but surprisingly understudied, idea that hormones and behaviour interact and feedback on one another, such that any one-directional relationship is overly simplistic [20]. This leads to the third problem of limiting ourselves to a challenge hypothesis framework.

### 2.3 Not all males are equal or equally likely to engage in challenges

Although male contests necessarily involve two (or more) males, the majority of research on the challenge hypothesis ignores how androgen profiles may influence the likelihood of males engaging in aggressive contests in the first place (although bystander androgen levels have been examined [21]). It stands to reason that not all challengers represent the same degree of threat, and thus, a challenger's relative competitive ability may not only predict *how* a challenged male responds (physiologically or behaviourally), but also predict *whether* he responds at all. For example, in an experimental study on wild chacma baboons (*Papio ursinus*), Bergman *et al.* [22] found that subject males only moved away from a ‘rival’ male (simulated using vocalizations played from a hidden speaker) when *both* males had high androgen levels. Indeed, androgen levels trumped other variables known to predict aggression in chacma baboons, such as dominance rank and rank disparity [23]. These results suggest that not all males may perceive themselves to be rivals—and that the hormonal profiles of males prior to contests may indeed provide valuable insights into what may ensue.

Here, we test the predictions of the challenge hypothesis while addressing each of these shortcomings using a dataset from a wild primate, the gelada (*Theropithecus gelada*). Geladas are an ideal model species for several reasons. First, gelada males have conspicuous social challenges where contestant males have clear roles (offence or defence) and the outcome of contests is unambiguous. Geladas are large-bodied, terrestrial primates that live in an open grassland [24,25], making it relatively easy to observe fights between rival males. These male fights mediate access to reproductive females that reside in harems (‘reproductive units’ [26]). Reproductive units comprise one harem-holding male (‘leader male’), 1–12 related females and their offspring, and occasionally one or more subordinate males (‘follower males’). Leader males engage in little to no parental care and mate with females throughout the year. Follower males are generally recently deposed leader males, but can also be young adult males that enter a harem as subordinates [27]. The leader male accounts for all reproduction in reproductive units with one male (‘one-male units’); however, follower males sire approximately 17% of offspring in multi-male units [26]. Leader males (often joined by follower males) fiercely guard their harems from ‘bachelor males’ that reside in all-male groups [24]. Importantly, bachelor males gain reproductive access to females only if they challenge and defeat a leader male (‘takeover’), or submissively enter as a follower [27]. During male contests, bachelor males are always on the offensive (i.e. they are the challenger males), and leader males are always on the defensive (i.e. they are the challenged males). Moreover, the winner always assumes the dominant leader male position, and the loser (if he survives) remains in the harem as a subordinate follower male or returns to a bachelor group.

**Figure 1.**
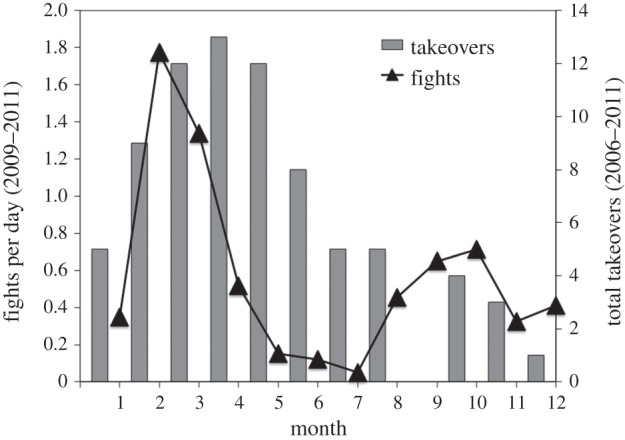
Male takeovers and fights. A histogram of successful takeovers across months of the year (grey bars) and the rate of male fights per month for the population of geladas living in the Simien Mountains National Park, Ethiopia. Successful takeovers exhibit a seasonal pattern, with most takeovers occurring during the end of the dry season.

Second, geladas offer an excellent opportunity for examining both (i) broad androgen profiles surrounding seasonal challenges, as well as (ii) individual androgen profiles across specific social challenges. The frequency of gelada contests exhibits strong seasonality, with a clear ‘takeover season’ spanning from February to June each year (figure 1). However, in contrast with most avian studies where an increase in contests occurs simultaneously with an increase in food availability [28], the majority of gelada contests occur during a period when food availability is the lowest (the end of the dry season). As we will explain, this distinction is valuable for separating out condition-based increases in testosterone from other factors that elevate testosterone. Furthermore, observations of social challenges between males (i.e. fights and takeovers) are frequent occurrences in this population (*n*=77 total takeovers 2006–2011; 0.35 takeovers per unit per year for one-male units and 0.27 takeovers per unit per year for multi-male units) [26]. Specifically, we have hormone and behavioural data from 30 takeovers for the 6 year study period.

Third, in many cases, we have data for both males (the challenger and challenged male) across the contest. For some takeovers (*n*=7), we have data from both males both before and after the contest. For others (*n*=23), we have detailed data from one of the males before the contest and both of the males after it (unless one of the males is killed, *n*=6).

Using the challenge hypothesis framework, we made the general prediction that challenges will be associated with an elevation in testosterone. However, we extended this investigation to include several of the shortcomings outlined above. First, we predicted that (i) elevations in androgens will be associated with both seasonal challenges (i.e. the takeover season) and social challenges (i.e. specific contests between a bachelor and a leader male). Second, with respect to social challenges, we predicted that (ii) in the months before contests occur, bachelor males that go on to challenge leader males will have higher testosterone profiles than bachelor males that do not challenge; and similarly, leader males that are challenged by bachelors will have lower testosterone profiles than leader males that are not challenged. Although we have observed a few unsuccessful takeovers, they are extremely rare in this population. We therefore restricted our dataset to include only successful takeovers. Last, we predicted that (iii) after a contest, winners (i.e. bachelors that become leaders) will exhibit an increase in testosterone and losers (i.e. leaders that become followers) will exhibit a decrease.

## 3. Methods

### 3.1 Subjects and study site

Data were collected from a population of geladas living in the Simien Mountains National Park, Ethiopia as part of the long-term University of Michigan Gelada Research Project. Over the 6 year study period (2006–2011), we collected behavioural, demographic and hormonal data from all adult males from 21 reproductive units and 13 all-male groups (*n*=133 males; including leaders, followers and bachelors). Throughout, we refer to dominant leaders and subordinate followers as ‘unit males’ to distinguish them from ‘bachelor males’. This population has been under near-daily observation since January 2006; and all males are individually recognized and habituated to observers on foot.

**Figure 2.**
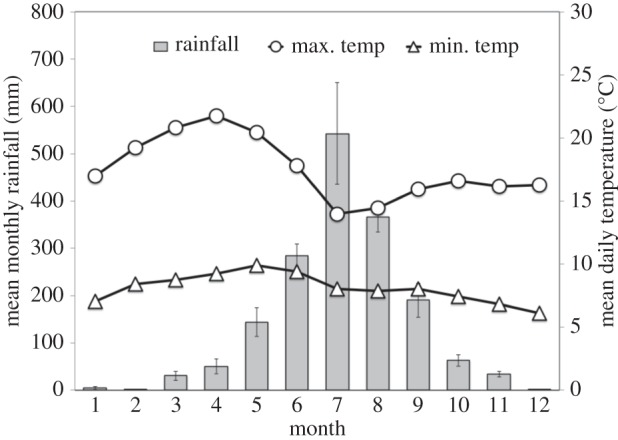
Seasonality of rainfall and temperature. Mean monthly rainfall (grey bars) and mean daily maximum temperature (white circles) and minimum temperature (white triangles) for the study site (data from 2006 to 2011). Error bars *represent*± s.e.*m*. (error bars on temperatures are smaller than the symbol).

The Simien Mountains National Park encompasses an area of Afroalpine habitat (150 km^2^, 3200–4500 m above sea level (a.s.l.)), including open grassland plateau and a few remnant forests. The region experiences pronounced ‘wet’ and ‘dry’ seasons each year [29,30]. The wet and dry seasons are variable each year, but generally occur during June–September (wet season mean monthly rainfall=310.8±30.3 mm; 2006–2011) and October–May (dry season mean monthly rainfall=38.0±7.6 mm; 2006–2011), respectively [29] (figure 2). Temperatures can approach freezing at night, but daily means range from 7.99°*C*±0.04 (mean minimum temperature, *n*=1843 days) to 17.66°*C*±0.07 (mean maximum temperature, *n*=1843 days). Rainfall and maximum and minimum temperatures are recorded on a daily basis using a rain gauge and digital thermometer centrally located in the gelada's home range [29].

### 3.2 Behavioural data collection

Status categories (leader, follower or bachelor) were determined by observations of group membership and dyadic dominance interactions. Among unit males, follower males are always subordinate to leader males, and (to the best of our knowledge) no dominance relationships appear to exist among leader males across reproductive units [24,31]. Therefore, we denote all dominant males ‘leaders’ and all subordinate males ‘followers’. Takeovers are conspicuous and (for the most part) discrete events where a bachelor challenges and subsequently overthrows a dominant leader male. After takeovers, former leaders have been observed to: (i) disappear (and presumably die, because we were unable to find these males during censuses of all surrounding groups), (ii) remain in the reproductive unit as a subordinate follower male, or (in rare cases) (iii) return to a bachelor group. A successful takeover ensued if the former leader exhibited submissive behaviours (e.g. fear barking, crouching, displacement, lip flips) towards the new leader (i.e. the former bachelor) and if the new leader copulated with females after the takeover.

Although the majority of males occupied only a single status category across the study (*n*=44 bachelors, *n*=10 followers, *n*=21 leaders), we observed several transitions between categories. Sixteen previously known bachelors became leader males, 22 leaders were deposed and became followers within their harem and five males transitioned from bachelor, to leader and finally to follower.

### 3.3 Faecal hormone collection and analysis

We collected faecal hormone samples in a targeted fashion from unit males; faecal samples were collected from leader (*n*=1376 samples) and follower males (*n*=684 samples) once per month across the entire study period (2006–2011). Faecal samples were collected from bachelor males (*n*=676 samples) opportunistically between 2006 and 2009 and 1× per male per month from 2010 to 2011. In total, we collected 2730 samples from 133 known males (approx. 14 samples per male; range 2–91 samples per male). In 2009, the manufacturer (Diagnostics Systems Laboratory) discontinued production of the testosterone antibody that we were using. Therefore, we had to employ two separate methods over the course of the study. In all analyses, we controlled for this variation and refer to these variables as ‘methods-based fixed effects’. Extraction and analysis of faecal testosterone (fT) metabolites and the validation of the old and new testosterone antibodies for use in geladas are described in the electronic supplementary material as well as elsewhere [12,29,32].

### 3.4 Do seasonal challenges influence male testosterone levels?

Given the seasonal pattern in gelada male contests (figure 1), it stands to reason that testosterone levels should rise for both the challenging bachelors and the challenged leaders during this period of increased competition. However, we needed to control for several factors that are known to affect male testosterone in other primates, such as weather, age and social status [33–36]. For rainfall, we designated each sample as a ‘wet’ or ‘dry’ season sample if the cumulative rainfall for the previous month was above or below the median (53.7 mm), respectively (*n*=1365 wet season, 1365 dry season). For temperature, we grouped samples based on whether they were collected during ‘hot’ days (max temperature>median maximum temperature, 17.5°C, *n*=397), ‘cold’ days (minimum temperature<median minimum temperature, 7.9°C, *n*=214) or ‘average’ temperature days (all other samples, *n*=2119). For age, we do not have known dates of birth for adult males, because gelada males disperse from their natal groups [37,38]. Thus, adult male ages were estimated to the nearest half year using secondary sexual characteristics such as canine eruption, tooth wear, pelage coloration and cape length [12,31]. For status, we split males into their broadest status categories (leader, bachelor, follower), and, to gauge overall competition across the population, we included the number of takeovers across our known harems for each month (range 0–5 takeovers per month).

These statistical analyses (and all subsequent analyses) were conducted in R (v. 3.0.3) [39]. Using the function ‘lmer’ in the lme4 package [40], we ran a linear-mixed model (LMM) with fT as the outcome variable to determine the effect of weather and social factors on male testosterone. We log-transformed the outcome variable (testosterone in ng g^−1^) in this and all subsequent analyses to approximate a normal distribution. Because each individual male had multiple data points (*n*=2730 samples from 64 leaders; 52 followers, 79 bachelors; range 2–91 samples per male), we included individual identity as a random effect. In addition to our methods-based fixed effects (see the electronic supplementary material) we included rainfall, temperature, male age, status and number of takeovers per month as additional fixed effects on testosterone levels. We compared univariate models that considered only a single fixed effect to multivariate models that considered a combination of fixed effects and/or interactions between fixed effects. We compared all candidate models using Akaike information criterion (AIC) and considered the model with the lowest AIC to be the best fit for our dataset [41,42]. If the difference in AIC was less than 2 for the lowest-ranked models, we considered both models to be equally good fits for the data [43]. For all LMMs, we visually inspected each model using a Q–Q plot, histogram of residuals and scatterplot of fitted versus residual values. For all models, residual values were normally distributed.

### 3.5 Do social challenges influence male testosterone levels?

According to the challenge hypothesis, we expected both the challenging bachelor and the challenged leader to exhibit elevated testosterone during and immediately after a takeover (the day of the takeover, plus the subsequent 6 days to increase our sample size; hereafter ‘week of takeover’). We fit an LMM of male testosterone using residuals calculated from an LMM of all male testosterone including only methods-based fixed effects. Because this elevation in testosterone is short-term, variation owing to age or seasonality should be negligible. We used these residuals as the outcome variable of a reduced model (*n*=126) and included the time period relative to takeover (three months prior to takeover or week of takeover) and the outcome of the challenge (winner or loser) for each male in the model (*n*=29), as well as an interaction between outcome and time period.

### 3.6 Before a social challenge, do testosterone levels of contestants predict upcoming challenges?

If a male's testosterone profile is associated with an upcoming challenge, we expected to observe differences among males before the takeover occurred. We predicted that bachelors who challenge leaders (*n*=11) should have higher testosterone levels relative to bachelors that do not challenge leaders (*n*=34) in the three months preceding a takeover. Additionally, we expected that leaders which are challenged (*n*=14) should have lower testosterone relative to other leaders that are not challenged (*n*=45) during the three months preceding a takeover. Recall that we used successful takeovers only in these analyses. Because the majority of challenges in this population were successful (85.6%), a challenge is tantamount to an immanent win for a bachelor. We included the same fixed effects from the previous model (methods-based fixed effects, rainfall, temperature and age) and only included samples collected in the three months prior to a male either acquiring or losing leader status. We then conducted two separate LMMs: one for leaders (*n*=881 samples) and another for bachelors (*n*=462 samples). We predicted that in the three months preceding a takeover, soon-to-be winners (i.e. bachelors that become leaders) have elevated testosterone levels relative to other bachelors and soon-to-be losers (i.e. leaders that lose their dominance status) have lower testosterone levels relative to other leaders.

### 3.7 After a social challenge, do testosterone levels depend on the outcome?

According to the winner/loser effect, we expected the testosterone levels of losers (i.e. former leaders) to decrease after the contest and the testosterone levels of winners (i.e. former bachelors) to increase. However, we were unsure exactly when after the contest these differences would appear. Therefore, we conducted another LMM using testosterone (log-transformed) as the outcome variable and this time included days after takeover (a continuous variable) and outcome (winner/loser) as additional fixed effects. In addition to these new fixed effects, we also included previous methods-based, temperature and rainfall effects (see the electronic supplementary material). We excluded age because of its strong correlation with days after takeover. We included only hormone samples (*n*=601) from the first year post-takeover for both winners and losers (excluding the first week post-takeover). For both this and the previous analysis, we included only winners that had tenure lengths of at least 1 year.

## 4. Results

### 4.1 Do seasonal challenges influence male testosterone levels?

**Figure 3.**
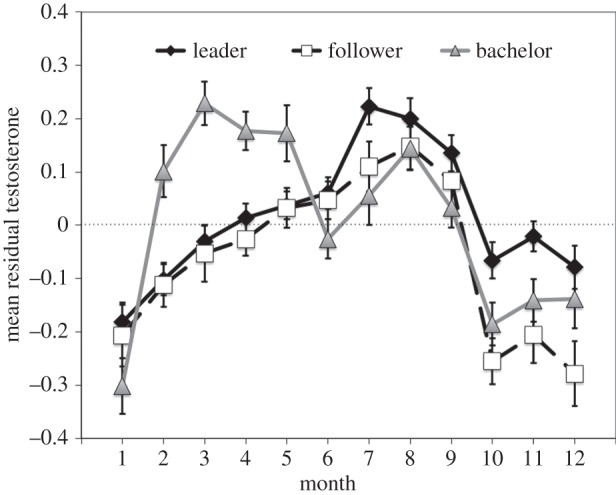
Seasonality of testosterone. The effect of male status and seasonality plotted against mean residual testosterone (±s.e.m.; controlling for age and methods-based effects). Leader and follower male testosterone strongly tracks patterns of rainfall with peak testosterone levels occurring at the end of the wet season. Bachelor testosterone exhibits a secondary peak during the time of increased takeovers, the ‘takeover season’.

A multivariate model that included all fixed effects and an interaction between takeovers per month and male status had the lowest AIC among all our candidate models (all *ΔAIC*>20). After controlling for methodological variables (antibody and wash), this interactive model yielded several significant associations. Most importantly, all males exhibited elevated testosterone during months of high takeovers (*β*=0.058, s.e.=0.010, *t*=5.55, *p*<0.01). Moreover, a significant interaction between number of takeovers and status indicates that unit males exhibit lower testosterone during months of increased takeovers (leader×takeovers per month: *β*=−0.063, s.e.=0.012, *t*=−5.10, *p*<0.01; follower×takeovers per month: *β*=0.038, s.e.=0.014, *t*=−2.68, *p*<0.05; figure 3).

Additionally, higher testosterone was associated with warmer days (hot days: *β*=0.103, s.e.=0.026, *t*=4.97, *p*<0.01), more rainfall (wet season: *β*=0.185, s.e.=0.015, *t*=12.28, *p*<0.01), younger age (*β*=−0.066, s.e.=0.007, *t*=−8.80, *p*<0.001) and leader male status (*β*=0.116, s.e.=0.033, *t*=3.56, *p*<0.01).

### 4.2 Do social challenges influence male testosterone levels?

**Figure 4.**
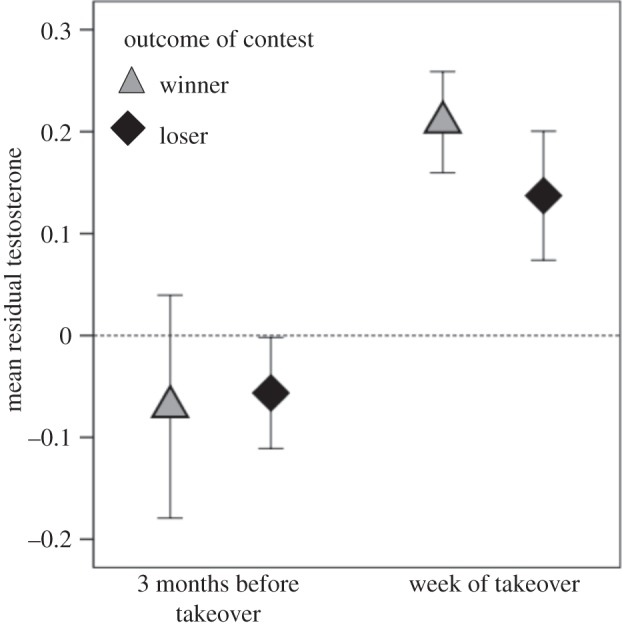
Testosterone levels of males across a takeover. Mean residual testosterone (±s.e.m.) for males involved in takeovers (controlling for methods-based effects). Both winners (bachelors that became leaders) and losers (leaders that lost their unit) exhibited a significant rise in testosterone during the week of takeover (i.e. the day of the takeover plus the following 6 days) compared with baseline levels (i.e. the average from the previous three months).

As predicted, both the challenging bachelor and challenged leader male's testosterone was elevated during the week of a takeover (*β*=0.231, s.e.=0.065, *t*=3.57, *p*<0.0001; figure 4). The testosterone residuals for winners and losers were indistinguishable both before the takeover and during the week of takeover (*β*=0.124, s.e.=0.079, *t*=01.57, *p*=0.12; figure 4).

### 4.3 Before a social challenge, do testosterone levels of contestants predict upcoming challenges?

**Figure 5.**
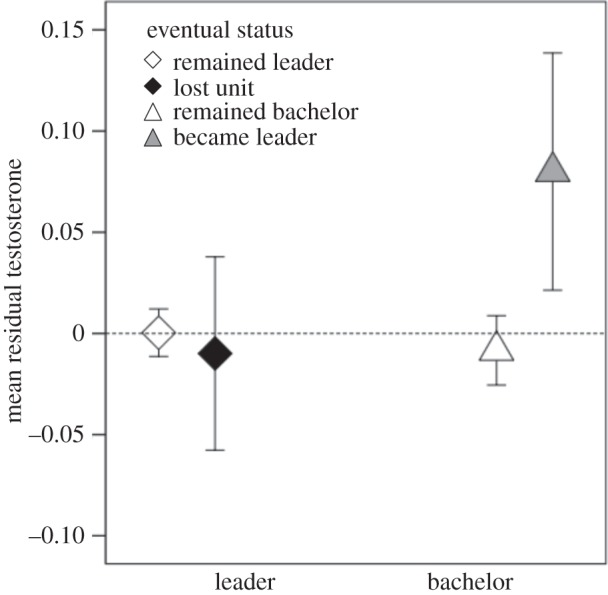
Testosterone levels of males leading up to a takeover. Mean residual testosterone (±s.e.m.; controlling for age, temperature, rainfall and methods-based effects) for males based on whether or not they lost their reproductive unit (for leaders) or became a leader (for bachelors) within the next three months. No difference was observed between leaders that lost their harem versus leaders that remained leaders (white and black diamonds). Bachelors that became leaders exhibited significantly higher testosterone than bachelors that remained bachelors (white and grey triangles).

After controlling for variables demonstrated to influence testosterone (see the previous result), testosterone levels of bachelors, but not leaders, predicted upcoming challenges. Bachelors that eventually became leader males within the next three months had elevated testosterone levels relative to other bachelors (*β*=0.142, s.e.=0.072, *t*=1.99, *p*<0.05; figure 5). No difference was observed between leaders that eventually lost their harem (within the next three months) and those that held on to their harem (*β*=0.058, s.e.=0.079, *t*=0.734, *p*=0.46; figure 5).

### 4.4 After a social challenge, do testosterone levels depend on the outcome?

Both the outcome of the takeover (winner/loser) and the length of time (number of days post-takeover) influenced male testosterone. Male testosterone for both winners and losers was negatively associated with the number of days after a takeover (*β*=−0.001, s.e.=0.0002, *t*=−6.92, *p*<0.001). In other words, although the takeover itself was associated with an immediate elevation in testosterone for both contestants, these levels decreased steadily as time passed. After controlling for this steady decline in testosterone, winners exhibited higher testosterone than losers (*β*=0.146, s.e.=0.045, *t*=3.23, *p*<0.01). This finding agrees with the results generated from the first LMM above; mainly, that leader males have higher testosterone than bachelors overall.

## 5. Discussion

Although geladas are not classified as seasonal breeders [44,45], there is nonetheless a clear seasonal bias to male contests that escalate to takeovers (figure 1). Remarkably, this unambiguous ‘takeover season’ was associated with relatively high testosterone levels for challenger males (i.e. bachelors) yet relatively low levels for the males challenged (i.e. leader males; figure 3). Zooming in on the individual hormone profiles during the weeks surrounding each takeover, both contestants' testosterone levels increased (relative to their own baseline from the previous three months) during the contest itself (figure 4). In short, the seasonal testosterone levels of leader males were *not* representative of their physiological maximum because they were subsequently able to boost testosterone during immediate social challenges. In summary, social challenges in male geladas appear to support the predictions of the challenge hypothesis, but seasonal ones do not—a pattern exactly opposite that reported for many birds [13] and at least one primate [46]. The question, then, is why? Why do leader males fail to ‘rise to the challenge’ of a predictable takeover season?

### 5.1 Leader males fail to rise to the challenge

One answer (not yet tested) is that, at the end of a very long dry season, leader males are simply unable to maintain high testosterone levels. Under this hypothesis, low testosterone among leader males might result from poor body condition (‘poor condition hypothesis’). High testosterone levels are metabolically costly to maintain, therefore males in poor condition may not be able to upregulate testosterone in response to seasonal challenges [47,48]. The gelada diet shifts from above-ground resources during the rainy season (mainly grass blades) to below-ground resources during the dry season (mainly corms) [30]. It is possible that this dietary shift leads to an energy shortfall that precludes optimal competitive performance, as measured by testosterone [49]. Bachelor males, on the other hand, may be able to offset this energy shortfall during the dry season because they do not spend any time/energy on costly mate guarding behaviours (e.g. yellow baboons, *Papio cynocephalus*, [50]). Moreover, bachelor males typically forage in smaller groups (the all-male group), which may allow them to travel less to gain sufficient nutrition [27]. Thus, males in different life-history stages may experience nutritional challenges differently across the year.

This poor condition hypothesis is partially supported by evidence that leader male testosterone is positively correlated with rainfall (Pearson's *r*=0.14, *t*_1368_=5.23, *p*<0.001), but bachelor male testosterone was not (Pearson's *r*=0.05, *t*_674_=1.39, *p*=0.16). If subsequent data (such as urinary C-peptide levels, a biomarker of energy balance [51]) support the hypothesis that leader males are in negative energy balance during the dry season, this would suggest that the testosterone maximum for non-seasonally breeding species is a *condition-based maximum* that varies based on the resources available to different males at different times [52]. As such, the amplitude of the testosterone maximum is expected to be proportional to the duration of the ‘breeding period’. Because the duration of the breeding period for a non-seasonal species (such as geladas) is essentially a life-history stage (for gelada males, this is the duration of his tenure as leader male, which can be several years [26]); thus, maintaining a testosterone maximum throughout this entire period may be physiologically impossible. Nevertheless, when leader male testosterone exhibits a seasonal nadir, bachelor males are quick to challenge them.

Our observations suggest that a challenge from a bachelor probably marks the end of a leader's tenure. Once a leader is targeted, he stands very little chance at success, because generally these attacks involve multiple bachelors chasing and fighting the leader until the point of exhaustion. Eventually, one of these bachelors wins the contest. Even if one bachelor fails, another bachelor quickly swoops in to finish the job. In short, leader males rarely succeed. Therefore, the optimal strategy for gelada leader males is to avoid being targeted by bachelors in the first place. How do they go about doing this? There are several possibilities. First, previous research indicates that leaders of multi-male units are less likely to be targeted by bachelors than one-male units [26]. In these multi-male units, leaders and followers together help to chase away bachelors that encroach on the harem [26]. Second, similar to predator–prey interactions, leader males may avoid being targeted by bachelors through ‘selfish herding’ with other harems [53]. Third, leader males may deter challenges through two putative sexually selected signals: chest patch colour and vocal displays [24,31]. The intensity, quality or frequency of such signals should indicate the fighting ability of their bearer [54]. Geladas are unique among primates in having a red patch of highly vascularized skin on their chest and neck; and previous work has suggested that leader males with larger harems have redder chest colour [31]. Several other primate species have demonstrated that red coloration in primate skin is mediated by levels of testosterone [55,56]. Additionally, gelada leader males perform conspicuous displays for bachelor audiences that culminate in a series of loud calls [27,57]. Early work from geladas reported that leader males who display more frequently were less likely to be targeted by bachelors [27]. And, evidence from Thomas langurs (*Presbytis thomasi*) [58] and chacma baboons [59] indicates that vocal displays are also testosterone-mediated (and also a possibility for gelada display vocalizations).

However, we were unable to detect any testosterone differences between the leader males that were targeted for takeovers and those that were not (figure 5). Although testosterone often mediates sexually selected signals that predict competitive ability [60–63], geladas may have other constraints that limit their fighting ability. For example, geladas live in hypoxic high-altitude habitats (this population lives at 3500 m a.s.l.), where available oxygen is only about 66% that at sea level (505 mmHg barometric pressure) and temperatures can routinely dip below freezing [29]. Given the vascularized nature of the gelada chest patch, chest redness could serve as an honest signal mediated by the trade-off between heat loss and advertising increased blood oxygen content [29].

### 5.2 Bachelor males rise to the challenge

By contrast, we found that all bachelors had elevated testosterone during the takeover season. All takeovers themselves were preceded by chases that involved the doomed leader male as well as all the bachelors in one or more all-male groups. Thus, although only a few bachelors participated in the actual fights (and takeovers), all bachelors (with only a few exceptions) participated in the chaos and chases leading up to a takeover. Thus, nearly all bachelors participated in some aspects of male contests even if they themselves did not challenge a leader male. This participation may have been enough to elevate the testosterone of all bachelors relative to leader males [64].

Moreover, we uncovered a significant difference between the bachelors that went on to challenge leaders when compared with those that did not. Male reproductive success for a gelada male is contingent upon him challenging and defeating a leader male within a reproductive unit (although he could enter the harem as a subordinate follower and achieve some matings [26]). Therefore, testosterone levels in a bachelor appear to predict his future trajectory. These results parallel those from related species, such as chacma baboons (*P. ursinus*) [65] and mandrills (*Mandrillus sphinx*) [66]. In these species, males ascending the dominance hierarchy have elevated testosterone [65,66]. Similarly, male grey-headed flying foxes (*Pteropus policephalus*) exhibiting high testosterone levels prior to experimental removal were more likely to re-establish a harem after reintroduction to their territory than their lower testosterone counterparts [67]. Taken together, these results suggest that elevated testosterone prior to a challenge may influence not only whether a contest occurs, but also the outcome of the contest itself.

In conclusion, we would like to return to the points raised in the Introduction. Had we examined only seasonal challenges or only social challenges, we would have reported either that the testosterone data from geladas fails to support the challenge hypothesis (for the former) or supports the challenge hypothesis (for the latter). Yet, really the most interesting story for geladas emerges when we examine the bachelors and leaders separately. Not only does the testosterone of challenging bachelors predict their future status (with higher testosterone characterizing the bachelors that eventually take over harems), but also the testosterone differential between leaders and bachelors predicts the unusual spike in takeovers at the end of the dry season.

## Supplementary Material

ESM - Method validation.doc. This is a Word file that provides a description of both the analytical and biological validation for a new testosterone antibody for use in gelada fecal samples

## Supplementary Material

 ESM - Fig 2 data.csv. This file provides the data used to construct Figure 2

## Supplementary Material

ESM - Fig 3 data.csv. This file provides the data used to construct Figure 3

## Supplementary Material

ESM - Fig 4 data.csv. This file provides the data used to construct Figure 4

## Data Accessibility

Both seasonal and social challenge testosterone data have been uploaded online as the electronic supplementary material.
