# Supplementary material for: Harem-holding males do not rise to the challenge: androgens respond to social but not to seasonal challenges in wild geladas
Source: R Soc Open Sci. 2014 Sep 24;1(1):140081. doi: 10.1098/rsos.140081 (PMC4448764; doi:10.1098/rsos.140081)
Supplement: ESM - Method validation.doc. This is a Word file that provides a description of both the analytical and biological validation for a new testosterone antibody for use in gelada fecal samples [file rsos140081supp1.docx]

**Supplemental Information**

*Fecal hormone collection and analysis*

We collected a pea-sized aliquot of fecal sample (mixed thoroughly) in 3 ml of a methanol:acetone solution (8:2). Samples were vortexed and later filtered and extracted using a solid-phase cartridge. All samples were washed with 2.0 ml of 0.1% sodium azide (NaN_3_) solution (2006-2010) or 20% MeOH (2011), placed in a sterile Whirl-pak bag with a silica dessicant, and stored frozen until shipment to the Core Assay Facility at the University of Michigan for radioimmunoassay (RIA). Dry fecal weights from all samples were obtained to the nearest +/- 0.0001 g, and hormones values were calculated as ng/g dry feces. Samples (N=2730) were run in duplicate across 57 assays (DSL=33, Pantex=24) at the Core Assay Facility.

*Fecal hormone analysis*

Beehner et al., (2009) previously validated a testosterone antibody for use in geladas (Diagnostics Systems Laboratory (DSL); Beckman Coulter). However, the manufacturer discontinued the DSL testosterone antibody at the end of 2009, and we continued analyses with a new testosterone antibody (Pantex testosterone direct I^125^kit, Santa Monica, CA) and a different wash step (20% MeOH used for 2011 only) for the solid-phase extraction. Therefore, it was necessary first to validate this antibody both analytically and biologically for use in geladas and second to control for the new Pantex testosterone antibody and methanol wash (20%).

*Analytical validation*

The Pantex antiserum is known to cross-react with testosterone (100%), 5α-dihdryotestosterone (6.9%), androsterone (0.52%), and anadrol (0.15%). First, a dose-response curve using a serially-diluted adult male fecal pool was shown to be parallel to a standard curve. Second, we spiked 9 standards with a low sample (12.5 μl) and a high sample (100 μl) and found a strong linear relationship between expected and observed values for both low (y=1.21x+0.36, R^2^=0.996, P<0.0001) and high (y=1.63+4.65, R^2^=0.986, P<0.0001) spiked samples. The sensitivity of the Pantex antibody was 0.20 ng/ml. Third, we used a low (~20% binding) a mid (~50% binding) and a high (~80% binding) fecal pool control in all assays. The respective inter-assay coefficients of variation were 28.9% (low), 13.3% (mid), and 15.1% (high). Our intra-assay coefficient of variation was 4.0% (N=6 samples at 100 μl).

*Biological validation*

For our biological validation we adhered to the same method used in Beehner et al., (2009). We assayed males of different age classes (N=16 juveniles, 30 prime-aged, and 12 late prime males) in a single assay (to control for inter-assay variation) to examine whether prime-aged males exhibit the highest testosterone values. As predicted, results indicated an inverse-U shape between age and testosterone. Juveniles and late-prime males exhibited the lowest testosterone levels, while prime-aged males exhibited the highest (Kruskal-Wallis: χ^2^=39.77, df=2, P<0.0001).

*Controlling for different methods*

To correct for variation in methods, we included two separate fixed effects in all of our linear mixed models: “antibody” (DSL or Pantex) and “wash” (NaN_3_ or 20% MeOH). The effect of each of these methods-based fixed effects was antibody (Pantex: β=-0.411, s.e.=0.07, t=-17.79 P<0.001) and wash (NaN_3_: β=-0.109, s.e.=0.02, t=-5.00, P<0.001) respectively.
